# Clinical Efficacy of Cysteamine Application for Melasma: A Meta-Analysis

**DOI:** 10.3390/jcm13237483

**Published:** 2024-12-09

**Authors:** Bing-Qi Wu, Yen-Jen Wang, Chang-Cheng Chang, Tzong-Yuan Juang, Yung-Hsueh Huang, Ying-Chuan Hsu

**Affiliations:** 1 Department of Education, China Medical University Hospital, Taichung 404327, Taiwan; jacky870914@gmail.com; 2 School of Medicine, College of Medicine, China Medical University, Taichung 40402, Taiwan; 3 Department of Dermatology, MacKay Memorial Hospital, Taipei 104217, Taiwan; yenjen4208@gmail.com; 4 Department of Cosmetic Applications and Management, MacKay Junior College of Medicine, Nursing, and Management, New Taipei City 11260, Taiwan; 5 Division of Plastic and Reconstructive Surgery, Department of Surgery, China Medical University Hospital, China Medical University, Taichung 404327, Taiwan; 6 Department of Cosmeceutics, China Medical University, Taichung 40402, Taiwan; tyjuang@mail.cmu.edu.tw; 7 Aesthetic Medical Center, China Medical University Hospital, Taichung 404327, Taiwan; 8 20skin Four Seasons Clinic, Taichung 408, Taiwan; dr.beaute@gmail.com (Y.-H.H.); dr.skin@yahoo.com.tw (Y.-C.H.)

**Keywords:** cysteamine, melasma, hydroquinone, tranexamic acid mesotherapy, modified Kligman’s formula

## Abstract

**Background:** Melasma is a challenging, acquired hyperpigmentary disorder. The gold standard treatment is Kligman’s formulation, which contains hydroquinone, tretinoin, and dexamethasone, but its long-term use is limited by the risk of exogenous ochronosis. Cysteamine, a tyrosinase inhibitor, reduces melanocyte activity and melanin production, showing strong depigmenting effects in patients resistant to Kligman’s formulation. Nonetheless, clinical studies have yielded inconsistent efficacy results. This meta-analysis aimed to assess the efficacy of cysteamine in treating melasma and to identify potential factors that may impact its therapeutic outcomes. **Methods:** A systematic search of PubMed, Embase, Web of Science, and CENTRAL, from the earliest record until August 2024, was conducted. Randomized controlled trials and quasi-randomized design studies related to topical cysteamine on melasma patients were included. The primary outcome was MASI or mMASI assessment after treatments. The current meta-analysis was conducted with a random-effects model. Subgroup analyses and meta-regressions were performed based on baseline MASI, disease duration of melasma, patient age, and sample size of the included studies. Funnel plots and Duval and Tweedie’s trim and fill method were adopted to assess the publication bias. **Results:** Eight studies were included for quantitative analysis. The analysis of MASI after topical cysteamine demonstrated a significant decrease compared to the placebo (*p* = 0.002). Compared to other melasma treatments, cysteamine did not show superior efficacy in mMASI (*p* = 0.277). The treatment efficacy of hydroquinone, modified Kligman’s formula, and tranexamic acid mesotherapy for melasma was not statistically different when compared to cysteamine (*p* = 0.434). Further analyses showed no benefit when allowing extended cysteamine application time (*p* < 0.0001). The meta-regression revealed the efficacy of cysteamine decreased as the duration of melasma increased (coefficient = 0.38, *p* = 0.0001, R^2^ = 0.99). The funnel plot displayed some asymmetry. The trim and fill method suggested the adjusted effect size was 0.607 (95% CI = −0.720 to 1.935). **Conclusions:** Cysteamine exhibited efficacy in treating melasma patients; however, its depigmentation effect was comparable to hydroquinone-based regimens, tranexamic acid mesotherapy, and modified Kligman’s formula. Using cysteamine in patients with a short duration of melasma may result in better efficacy.

## 1. Introduction

Melasma impacts individuals across all racial and ethnic backgrounds, with a higher prevalence among women and those with darker skin tones [1]. The development of melasma is intricate, involving various causative factors, such as exposure to ultraviolet radiation, genetic susceptibility, pregnancy, and the use of external hormones [2].

The established gold standard treatment for melasma is Kligman’s formulation, which comprises 5% hydroquinone, 0.1% tretinoin, and 0.1% dexamethasone. The side effects include erythema, desquamation, and steroid-induced telangiectasia. Combinations having higher-potency corticosteroids, like mometasone, are marketed as ‘modified Kligman formulation’ (mKF). However, the risk of exogenous ochronosis still precludes its long-term use.

Cysteamine was initially identified in the mid-20th century as a component of the coenzyme A metabolic pathway, an aminothiol derived from the natural degradation of L-cysteine [3,4]. Cysteamine inhibits tyrosinase activity and decreases melanocyte hyperactivity, leading to decreased melanin production [5,6]. The exact mechanism by which cysteamine inhibits melanogenesis remains incompletely understood [7,8]. However, it is known to increase intracellular glutathione levels, which shifts melanin synthesis from eumelanin to pheomelanin [9,10]. Additional mechanisms may involve the thiol nature of cysteamine. Thiol-based depigmenting agents are recognized as inhibitors of tyrosinase and peroxidase, two key enzymes in melanin biosynthesis [11]. Moreover, thiols are capable of scavenging dopaquinone, thereby removing it from the melanogenesis pathway [12]. These molecules may also act as chelators of iron and copper ions, disrupting Fenton reactions that contribute to pigment synthesis [13]. As for the associated side effects, those from cysteamine are generally well tolerated and include erythema, dryness, itching, burning sensation, and irritation. Among these, itching and burning are the most frequently reported adverse effects [8,14,15]. However, most side effects are transient, typically subsiding significantly within approximately one week after treatment initiation and gradually resolving over time [8]. Its potent depigmenting effect has been evidenced in melasma patients who have shown resistance to Kligman’s formula [16]. Recent randomized, double-blind, placebo-controlled studies have also demonstrated its therapeutic efficacy [8,14]. Nevertheless, clinical investigations comparing its efficacy to mKF have yielded inconsistent results [15]. A meta-analysis concluded that 5% cysteamine is effective in the treatment of melasma, with a low incidence of side effects or adverse reactions [17]. Recently, a complex based on cysteamine has been proposed as a potentially viable alternative to mKF for long-term use [18]. This study aims to perform a meta-analysis to investigate the effectiveness of cysteamine in the treatment of melasma and to identify factors that could influence its therapeutic results.

## 2. Methods

### 2.1. Search Strategy and Study Eligibility

This research adhered to the guidelines established by the Preferred Reporting Items for Systematic Reviews and Meta-Analyses (PRISMA) [19]. Additionally, the study protocol was pre-registered with INPLASY (INPLASY202480016).

The PICO framework (population, intervention, comparison, and outcome) utilized in the present meta-analysis is delineated as follows: P: participants with facial melasma, I: topical cysteamine component treatment, C: no comparison restrictions, and O: mMASI (Modified Melasma Area and Severity Index) or MASI (Melasma Area and Severity Index).

A comprehensive electronic search was performed across various online databases, including PubMed, Embase, Web of Science, and CENTRAL, covering records from their inception until August 2024. Regarding the specific search methodology, two authors (B.-Q.W. and Y.-J.W.) independently executed electronic searches within the aforementioned databases, utilizing the following combination: (melanosis or melasma or chloasma) AND (cysteamine) AND (MASI OR mMASI). In the course of this investigation, two authors (B.-Q.W. and Y.-J.W.) conducted an initial screening of the identified titles and abstracts to determine their eligibility. Additionally, the reference lists of the identified review articles were examined [17]. Additional manual searches were also performed. In instances where a consensus could not be reached between the two authors, a third author (CCC) was consulted for further deliberation.

The following inclusion criteria were adopted: (1) randomized controlled trials (RCTs) or quasi-randomized controlled trials, (2) studies enrolling human participants, (3) studies evaluating melasma using MASI or mMASI, (4) trials encompassing topical cysteamine component intervention, and (5) studies evaluating the depigmentation effect at least 4 months or 16 weeks. This study imposed no language restrictions.

Abiding by exclusion criteria, this study excluded (1) animal studies, (2) studies lacking groups for comparison, (3) studies lacking outcome measures of MASI or mMASI, and (4) studies using cysteamine as one component in combination therapy for treating melasma. This study did not require informed consent or institutional review board approval because it did not involve patient-identifying data.

### 2.2. Appraisal of Methodological Quality

To evaluate the methodological quality of the included studies, we adopted the Cochrane risk of bias tool for randomized studies (version 2, RoB 2, London, United Kingdom) [20,21]. Compared to the Jadad scale, Rob 2 comprises six primary components for assessing the quality of studies: the randomization process, intervention adherence, missing outcome data, outcome measurement, selective reporting, and the overall risk of bias. The Cochrane Handbook advises the utilization of the RoB tool for the appraisal of studies when feasible [21]. Risk-of-bias visualization (robvis) was used to display the appraisal results [22].

Within the intervention adherence domain of the Risk of Bias 2 (RoB 2) framework, the authors opted for a per-protocol evaluation from the two available options for literature assessment: intention-to-treat (which pertains to intervention assignment) and per-protocol (which focuses on intervention adherence).

### 2.3. Data Extraction and Management

Two independent authors (B.-Q.W. and YHH) extracted data, including the first author, publication year, country, study design, patient age, Fitzpatrick skin type, baseline MASI, melasma duration, follow-up durations, melasma clinical assessment tools, and cysteamine regimens. We rigorously followed the guidelines specified in the Cochrane Handbook for Systematic Reviews of Interventions for data extraction, conversion, and merging of data from different study arms [23,24,25]. If the studies included both intention-to-treat and per-protocol outcomes, this study specifically extracted the per-protocol data. If post-treatment data were available at multiple time points, we extracted the outcome reported at the end of the intervention for statistical analyses.

### 2.4. Statistical Analyses

The current meta-analysis was conducted with a random-effects model using Comprehensive Meta-Analysis software (version 4, Biostat, Englewood, NJ, USA) [26]. A two-tailed *p*-value of less than 0.05 was established as the threshold for statistical significance.

Mean differences (MDs) and 95% confidence intervals (CIs) were utilized to estimate the efficacy of cysteamine usage. The calculation involved determining the post-intervention difference in MASI scores between the cysteamine-treated group and the control group. A negative effect size value indicates a beneficial outcome of using cysteamine. Heterogeneity was assessed through the application of I^2^ statistics and Cochran’s Q tests. I^2^ values were categorized as low, moderate, and high heterogeneity at thresholds of 25%, 50%, and 75%, respectively [27].

Subgroup analyses were performed to assess the impact of controlled type and different cysteamine application protocols on heterogeneity within the study. The distinction between subgroups was measured utilizing Cochran’s Q test. A *p*-value lower than 0.05 in Cochran’s Q test indicates statistically significant differences among the related subgroups. Meta-regression analyses were performed to explore additional covariates that interacted with the summary effect size, such as baseline MASI, disease duration of melasma, patient age, and sample size of the included studies.

To confirm the robustness of the results, one-study removal methods were employed to assess whether there was a statistically significant change in the summary effect size after removing a specific trial from the analysis [28].

The assessment for potential publication bias was conducted in accordance with the guidelines established in the Cochrane Handbook for Systematic Reviews of Interventions [29]. Funnel plots were generated and visually inspected for symmetry. Duval and Tweedie’s trim and fill method was adopted to estimate the number of missing studies [30]. Egger’s regression tests were performed when there were at least 10 datasets available.

## 3. Results

### 3.1. Study Characteristics

**Figure 1:**
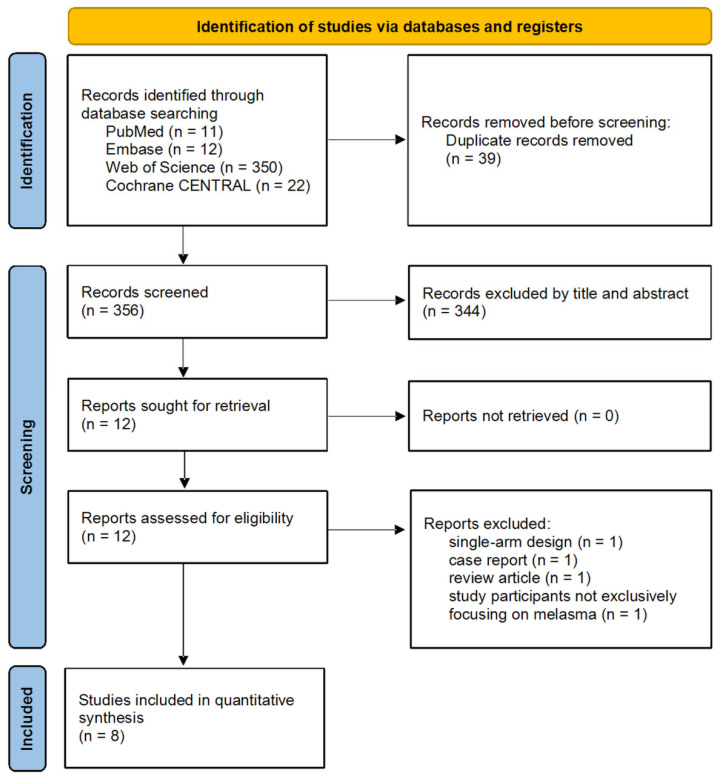
PRISMA 2020 flowchart for the current meta-analysis.

Initially, a total of 395 studies were identified. After the screening process, 12 studies were chosen for comprehensive evaluation of their full text. We excluded four studies after full-text evaluation. One study was excluded because it did not exclusively focus on patients with melasma. Another study was excluded due to its trial design, which lacked a control group (i.e., a single-arm study). One study was excluded as it was a review article, and another was excluded because it was a case report. Ultimately, eight studies were incorporated into our research [7,8,14,15,18,31,32,33] (Figure 1). The included studies were from Australia (*n* = 1), Brazil (*n* = 1), Iran (*n* = 5), and India (*n* = 1). Two studies had a placebo-controlled design [8,14]. Six studies compared cysteamine with other treatments for melasma [7,15,18,31,32,33]. In summary, a total of 415 patients were included in the study population (Table 1). The mean age ranged from 35.0 years to 43.1 years. The baseline MASI of participants ranged from 6.54 to 17.2. Disease duration varied between 3.41 and 12 years, with one study not reporting the duration. Two studies included patients with Fitzpatrick skin type II; three studies included Fitzpatrick skin type V patients; and one study did not reveal the Fitzpatrick skin type. The regimen details of the retrieved trials are summarized in Table 2. Regarding the cysteamine regimens, five studies standardized the application time of cysteamine to 15 or 30 min throughout their treatment protocol [15,18,31,32,33]. In one additional study, patients were permitted to administer cysteamine cream for up to 2 h if no skin irritation occurred [7]. Two studies did not report the exact cysteamine exposure time [8,14].

### 3.2. Quality Assessment and Risk of Bias

**Figure 2:**
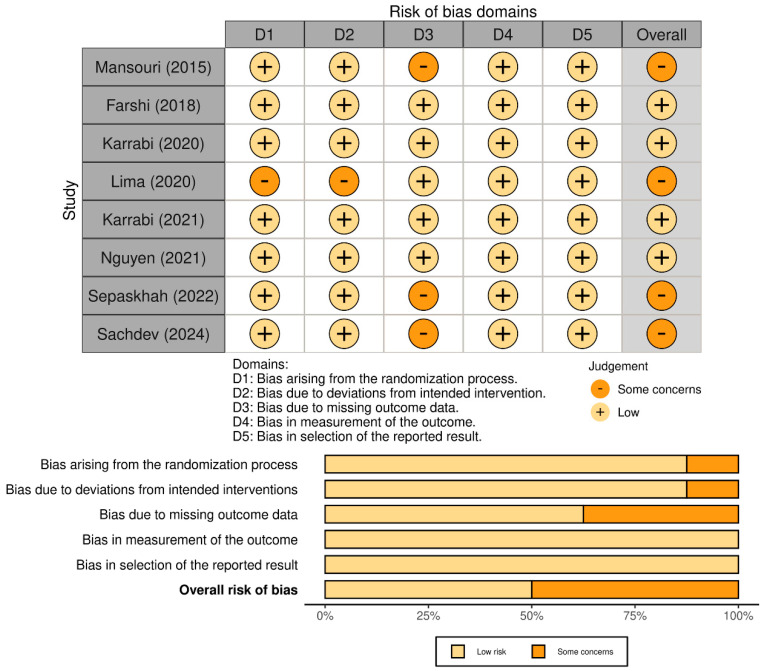
Summary of quality assessment for the studies included in the current meta-analysis using version 2 of the Cochrane risk-of-bias tool for randomized trials [7,8,14,15,18,31,32,33].

Among the eight recruited studies, four had a “low” risk of bias in the overall assessment (Figure 2). Other studies were categorized as having “some” risk of bias due to the following reasons: (1) the absence of a strict randomization process [7], (2) the exclusive reporting of per-protocol data without investigating the impact on the results due to missing data [8,18,33], and (3) allowing participants to extend the treatment exposure time without reporting the exact duration [7].

### 3.3. Effects of Cysteamine Utilization on Melasma

**Figure 3:**
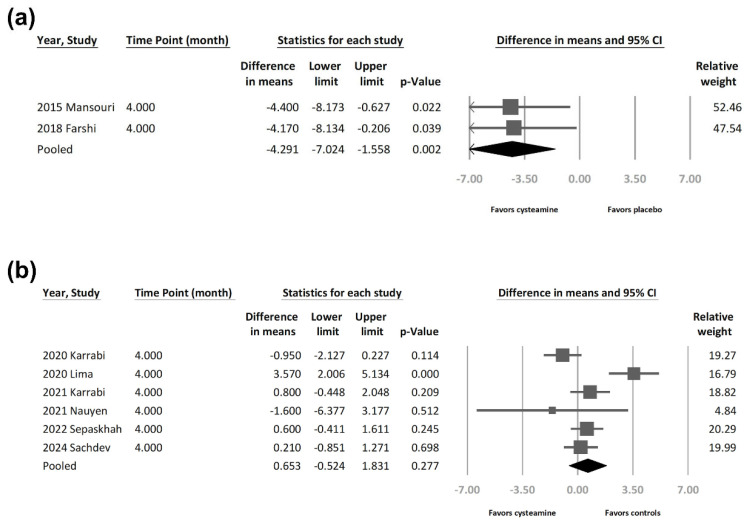
(**a**)**.** Forest plot presenting mean difference in depigmentation efficacy between cysteamine and placebo in patients with melasma. (**b**)**.** Forest plot presenting mean difference in depigmentation efficacy between cysteamine and other treatments in patients with melasma [7,8,14,15,18,31,32,33].

Based on two studies comparing cysteamine with placebo, cysteamine demonstrated superior efficacy in reducing skin hyperpigmentation in patients with melasma, showing a statistically significant effect (MD = −4.291 [95% CI = −7.024 to −1.558], *p* = 0.002, I^2^ = 0%) (Figure 3a).

**Figure 4:**
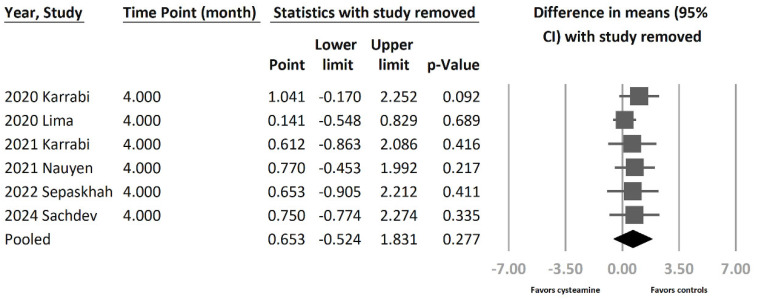
In the efficacy of cysteamine compared to other treatments, a sensitivity analysis was performed using the one-study-removal method. The main result remained consistent without significant changes after excluding any of the included trials [7,15,18,31,32,33].

Cysteamine demonstrated lower efficacy in reducing skin hyperpigmentation in patients with melasma based on six studies comparing cysteamine with other melasma treatments, though not reaching statistical significance (MD = 0.653 [95% CI = −0.524 to 1.831], *p* = 0.277, I^2^ = 77.2%) (Figure 3b). High heterogeneity was observed. To further ascertain the efficacy of cysteamine utilization, a sensitivity analysis was performed employing the one-study-removed method. The summary effect sizes remained consistent despite the exclusion of any of the included studies (Figure 4). The effect size showed nearly a 75% overall difference after stepwise removal of two studies. Removing the study by Lima et al. reduced the effect size from 0.653 to 0.141, while removing the Karabi study increased the effect size from 0.653 to 1.041. However, these new effect sizes remained within the confidence interval range of the original effect size.

**Figure 5:**
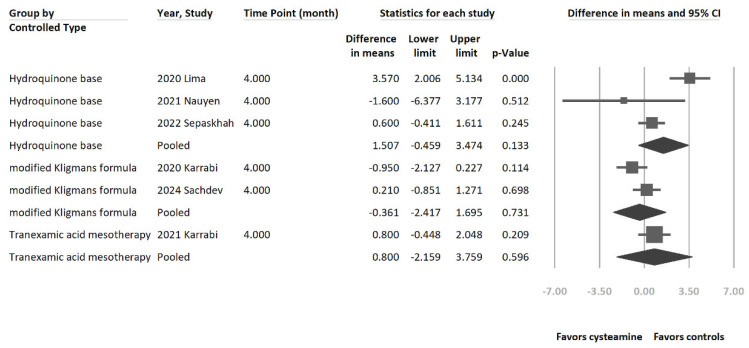
The forest plot of subgroup analysis using different melasma treatments versus cysteamine as the moderator. The treatment efficacy of hydroquinone, modified Kligman’s formula, and tranexamic acid mesotherapy for melasma was not statistically different when compared to cysteamine. The Cochran’s Q test for the effect sizes difference among melasma treatment subgroups was insignificant (*p* = 0.434) [7,15,18,31,32,33].

Subgroup analysis was performed to investigate the depigmentation effect between cysteamine and different melasma treatments (Figure 5). Studies were organized based on the treatments used in controlled groups, including hydroquinone-based therapy, tranexamic acid mesotherapy, and modified Kligman’s formula. None of the depigmentation effects reached the statistically significant difference among hydroquinone-based therapy (MD = 1.507 [95% CI = −0.459 to 3.474], *p* = 0.133, I^2^ = 82.3%), modified Kligman’s formula (MD = −0.361 [95% CI = −2.417 to 1.695], *p* = 0.731, I^2^ = 51.4%), or tranexamic acid mesotherapy (MD = 0.800 [95% CI = −2.159 to 3.759], *p* = 0.596, I^2^ = 0%). The Cochran’s Q test for the effect size differences among melasma treatment subgroups was insignificant (*p* = 0.434).

**Figure 6:**
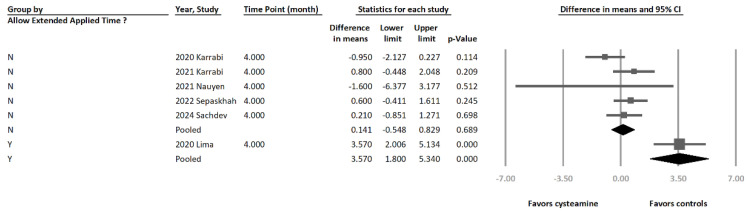
The forest plot of subgroup analysis is based on the permitted extended cysteamine application. In the study conducted by Lima et al., participants were asked to apply the cream for 15 min on the first night and gradually extend the duration up to 2 h. The Cochran’s Q test for the effect size differences among subgroups was significant (*p* < 0.0001) [7,15,18,31,32,33].

In another subgroup analysis, the studies were categorized as “Yes” or “No” based on whether the protocol allowed extended cysteamine application time (Figure 6). For the “No” subgroup [15,18,31,32,33], no significant difference was observed between cysteamine and controls (MD = 0.141 [95% CI = −0.548 to 0.829], *p* = 0.689, I^2^ = 29.6%). For the “Yes” subgroup [7], the treatment effect significantly favored other melasma treatments (MD = 3.57 [95% CI = 1.800 to 5.340], *p* < 0.0001, I^2^ = 0%). The Cochran’s Q test for the effect size differences among subgroups was significant (*p* < 0.0001).

**Figure 7:**
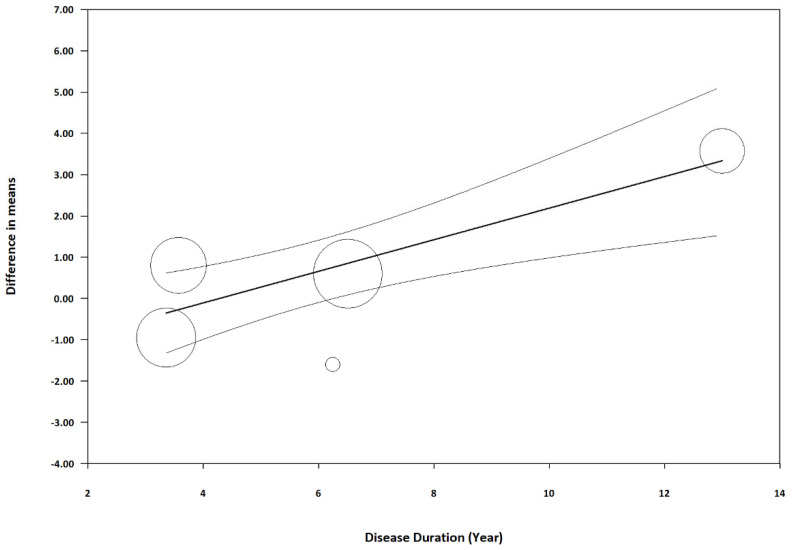
Meta-regression of difference in means on disease duration. The coefficient was 0.38 with *p* = 0.0001. Sachdev et al.’s study was omitted during the meta-regression due to the absence of exact disease duration in their trial.

Meta-regression analyses were performed to investigate the potential depigmentation effect between cysteamine and other melasma treatments by identifying covariates across the included studies. The analyses displayed that the disease duration of melasma (coefficient = 0.38 per year, *p* = 0.0001, R^2^ = 0.99) could modify the effect difference between cysteamine and other melasma treatments (Figure 7). A sensitivity analysis was conducted by removing Lima et al., and this covariate did not reach statistical significance (coefficient = 0.23 per year, *p* = 0.32). Other identified covariates, such as baseline MASI, patient age, and sample size, did not alter the difference in effect (Table 3).

**Figure 8:**
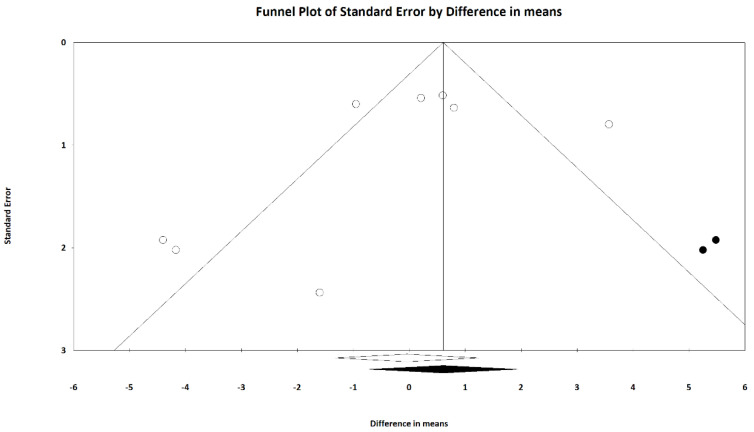
The funnel plot of included studies showed asymmetric distribution. The trim and fill method suggested the adjusted effect size was 0.607 (95% CI = −0.720 to 1.935).

The funnel plot (Figure 8) of the eight included studies for publication bias assessment displayed some asymmetry in effect size (MD) distributions. The trim and fill method suggested the adjusted effect size was 0.607 (95% CI = −0.720 to 1.935). Although the direction of the effect size changed, it did not reach statistical significance.

## 4. Discussion

This study represents the inaugural meta-analysis examining the comparative treatment efficacy of cysteamine to a placebo group or other treatments, including mKF, hydroquinone, a hydroquinone/ascorbic acid combination, and tranexamic acid mesotherapy. Though cysteamine demonstrated superior efficacy to placebo, the efficacy difference between cysteamine and other melasma treatments was not statistically different. The pooled treatment effect from current studies revealed no significant advantage of cysteamine over other treatments on melasma. The disease duration of melasma affects the efficacy of cysteamine: the treatment effectiveness decreases mMASI by 0.38 points as the disease duration increases by one year.

Cysteamine demonstrated some effectiveness compared to the placebo. Our findings align with a previous meta-analysis confirming the effectiveness of cysteamine [17]. Dos Santos-Neto et al. reported the efficacy after four months of cysteamine. However, the authors did not report the correlation coefficient, which is crucial for accurately determining the effect size [24]. Furthermore, it lacks comparisons of cysteamine with other treatments for melasma. Chang et al. reviewed and analyzed the efficacy and adverse effects of topical treatments for melasma [34]. They inferred that hydroquinone-containing agents and cysteamine had comparable efficacy, with moderate-to-high heterogeneity observed. To provide a clearer reference for clinical decision making and reduce the heterogeneity observed in previous studies, our research focused on comparing the depigmentation effects of cysteamine with those of other treatments. The main finding was also validated through sensitivity analysis to prevent the results from being overly influenced by a single study. In addition to comparing cysteamine with alternative melasma therapies, we also conducted subgroup analyses to evaluate whether differences in efficacy exist among various treatment modalities. This approach also lowered the heterogeneity. Another strength of our study is the application of meta-regression analysis, which allows for the identification of potential clinical or trial design factors that may influence outcomes across different studies, particularly continuous variables, such as patient age, baseline disease status, and sample size. When compared to other treatments, cysteamine may not have significant advantages in reducing MASI/mMASI. Although the effect size suggested that cysteamine could be less effective than other melasma treatments, this difference did not reach statistical significance. In Nguyen’s study, the confidence interval exhibited a significantly broader range compared to other studies, given a sample size of merely 10 participants in each study arm [32]. This led to a reduction in statistical power and an increase in the width of the pooled 95% confidence interval. The studies included in comparing cysteamine with other melasma treatments showed similar efficacies, except for Lime et al. In their research, cysteamine exhibited inferior performance compared to hydroquinone in depigmentation [7]. The differences observed compared to other studies may be attributed to the quasi-randomized and open-label study design. The relatively high heterogeneity within this subgroup has diminished the precision of the outcome. Further larger sample sizes or multi-center trials are anticipated to confirm the efficacy difference.

The treatment efficacy of hydroquinone, mKF, and tranexamic acid mesotherapy for melasma was not statistically different when compared to cysteamine. While the effect size direction favored cysteamine over mKF, the difference was not statistically significant. Two other subgroups exhibited an opposite effect size direction. Cysteamine was less effective compared to their control groups, which included hydroquinone-based treatments and tranexamic acid mesotherapy. While hydroquinone cream can be employed as a single treatment, several clinical trials have identified additional clinical efficacy resulting from the above treatments. Hydroquinone has the potential to be incorporated into a sequential treatment protocol, such as topical clobetasol, Danggui Shaoyao powder, and nitrogen plasma [35,36,37,38]. A meta-analysis has demonstrated that microneedling plus tranexamic acid could serve as a viable alternative for melasma treatment [39]. However, the depigmentation effect was only statistically significant at 4 weeks; the 16-week follow-up interval showed no significant difference compared to their control groups. Our subgroup analyses, which compared cysteamine and mesotherapy, indicated that neither the cysteamine nor the tranexamic acid treatment demonstrated superiority. Due to the limited research in this field, additional trials may be required to investigate the effectiveness and safety.

The subgroup analysis revealed that within a fixed cysteamine application time, cysteamine had comparable efficacy to other melasma treatments. However, in the subgroup allowed for extended exposure up to 2 h, the cysteamine group demonstrated less efficacy than other treatments for melasma. The effectiveness of cysteamine may depend on the duration of exposure. Only one study is available in this subgroup [7], which lacks specific details concerning the average duration of cysteamine use among participants or the number of participants who adhered to the extended application recommendations. Hence, the observation derived from this analysis may not conclusively establish that the duration of treatment significantly impacts the efficacy. Additional standardized dose–response trials are needed to validate these findings. In addition to implementing a standardized regimen, another direction for future research is to divide treatment groups into short-term and long-term follow-up based on a reasonable cut-off point. This approach would allow for the evaluation of the association between efficacy and side effects with prolonged exposure.

To the best of our knowledge, there is currently no literature, case report, or review discussing potential differences in cysteamine efficacy across different countries. Most of the included studies were conducted in Iran. We hypothesized that efficacy discrepancies might exist; however, our sensitivity analysis revealed no significant changes in the overall effect. Moreover, trials from other countries were limited to single studies, making subgroup analyses by country infeasible due to insufficient statistical power. A retrospective cohort study investigating racial differences in melasma treatment utilization found no significant differences in treatment rates among various racial groups, including Hispanic, White/Caucasian, Asian/Pacific Islander, and African American/Black individuals [40]. These findings suggest that cysteamine may have similar efficacy across different races and ethnicities. However, it is notable that the cohorts in this study did not include Persian or Arabian populations. Therefore, the generalizability of these findings underscores the need for large-scale cohort studies that focus on these specific racial and ethnic groups.

According to previous studies, cysteamine exhibited a lower incidence of side effects, with most being reported as mild or moderate in nature [15,17,32]. The most commonly reported side effects of cysteamine include erythema, a burning sensation, pruritus, and skin dryness, with an incidence rate of 42.2% as reported by Chang et al. [34]. This rate is even lower than that of hydroquinone-containing combination therapy, which has an incidence rate of 50.9% [34]. Of note, adverse effects from cysteamine were observed to be transient and progressively diminished over the course of treatment, as documented in studies conducted by Farshi et al. and Mansouri et al. [8,14]. Subsequent research has corroborated these findings [7,15,31]. Particularly, two of the most recent trials reported solely the number of participants experiencing side effects, omitting a comprehensive safety evaluation in their study designs [18,33]. This trend suggests that cysteamine is increasingly recognized as a safer alternative to traditional therapies for the treatment of melasma. A possible challenge would be a noticeable odor when cysteamine is applied to the face. From our perspective, patients generally express greater concern regarding the side effects associated with combined hydroquinone use, such as ochronosis and skin atrophy, rather than the odor of cysteamine. This is likely because cysteamine is typically applied to the face for only 15 min during each use. We also noticed current clinical trials have reported the safety profile of cysteamine in a relatively subjective manner without clearly defining the ordinal cut-off points. For instance, Karrabi et al. used an ordinal scale—none, mild, moderate, and severe—based solely on patients’ subjective perceptions [15]. The lack of precise definitions for each category may hinder clinicians’ ability to accurately assess the safety profile. We recommend that future research adopt well-designed methodologies to investigate side effects, incorporating objective measures, such as follow-up photographs evaluated by blinded assessors.

The efficacy of cysteamine decreased as the duration of melasma increased when compared to other melasma treatments. This finding suggests that cysteamine offers more advantages in patients with shorter-duration melasma. In contrast, hydroquinone-based regimens, tranexamic acid mesotherapy, and modified Kligman’s formula have been shown to be more effective than cysteamine for longer-duration melasma. The R-square index of 0.99 suggests that the duration of the disease could account for the majority of the variances in efficacy observed in recent trials comparing cysteamine with other treatments (i.e., 99% of the total variance in true effects in depigmentation could be explained by applying disease duration as the covariate). However, Lima et al. reported a disease duration that was more than double that of other studies [7]. The coefficient became insignificant after we excluded this study. The current trials included comparable disease durations, indicating that disease duration did not significantly impact the efficacy of depigmentation. Currently, no specific literature has explored the relationship between cysteamine and disease duration, nor have any case reports suggested a reduced depigmentation effect in patients with longer disease durations. Cysteamine is recognized as a well-tolerated and effective alternative for treating mild to moderate melasma [41]. Our findings further suggest that clinicians should consider disease duration when prescribing cysteamine.

We observed potential publication bias across the included studies, which may stem from the comparable efficacy of the treatments examined. We speculated that the lack of substantial therapeutic superiority may reduce authors’ motivation to publish related findings. These studies concluded that cysteamine could serve as an effective treatment for melasma; however, they did not demonstrate superior effectiveness compared to other treatments. Additionally, the comparison groups achieved better outcomes in other aspects, such as the Melasma Quality of Life Scale (MELASQoL) [7]. Notably, these studies were not designed as non-inferiority trials. In certain instances, existing publication bias could significantly skew the conclusions of meta-analyses. In our current analysis, the direction of the effect size changed after applying the trim-and-fill method. Thus, interpretations of cysteamine’s efficacy should be cautiously approached, even though the adjusted effect size did not reach statistical significance. Future studies with larger sample sizes or well-designed, propensity-matched cohort analyses could help mitigate this bias and provide a more accurate assessment of cysteamine’s therapeutic effectiveness.

This study has several limitations. Firstly, multivariate regression models cannot be utilized due to the limited number of included studies, which hinders the ability to predict interactions among clinical factors. Secondly, eight trials were ultimately included in our analysis. The limited number of studies necessitates cautious interpretation of covariates beyond disease duration. While potential trends may be present within the current evidence, they may not have been detectable in our meta-regression analysis. Nevertheless, our study represents the first application of meta-regression analysis to identify potential clinical factors that influence the efficacy of cysteamine, thereby offering valuable insights into this field. Thirdly, most of the studies were conducted in Asia, particularly in Iran. Consequently, there is insufficient evidence to reliably extrapolate the findings to other continents based solely on our results. It is noteworthy that there is no article discussing the differences in treatment among various races and ethnicities. Lastly, the long-term effects of cysteamine treatment remain ambiguous according to our research findings. Existing randomized controlled trials have predominantly concentrated on treatment durations of up to 4 months, or 16 weeks. Importantly, a previous case report revealed a significant decrease in MASI scores after six months of treatment, which followed a crossover from Kligman’s formula to cysteamine cream [16]. It is plausible to expect that the depigmentation effects may endure with long-term use of cysteamine; however, this hypothesis necessitates validation through more robust clinical trials.

## 5. Conclusions

The most efficacious modality for melasma remains inconclusive. Cysteamine did not show superior efficacy when compared to hydroquinone-based regimens, tranexamic acid mesotherapy, or modified Kligman’s formula. Using cysteamine in patients with a short duration of melasma may result in better efficacy. As cysteamine did not demonstrate significantly superior efficacy over other treatments, clinicians should balance treatment efficacy, potential side effects, and long-term safety when making recommendations.

## Figures and Tables

**Table 1:** Characteristics of included studies.

Author	Year	Country	Trial Design	Study Population *	Fitzpatrick Skin Type	Mean Age (Year)	Baseline MASI (Mean)	Follow-Up (Month)	Disease Duration (Year)
Mansouri et al. [8].	2015	Iran	Randomized, double-blind, placebo-controlled	Cysteamine group (*n* = 28) Placebo group (*n* = 27)	III–IV	Cysteamine: 39.9 Placebo: 39.7	Cysteamine: 17.2 Placebo: 13	2, 4	Cysteamine: 5.9 Placebo: 6.2
Farshi et al. [14].	2018	Iran	Randomized, double-blind, placebo-controlled	Cysteamine group (*n* = 20) Placebo group (*n* = 20)	III–IV	Cysteamine: 39.9 Placebo: 39.7	Cysteamine: 18.1 Placebo: 13.2	2, 4	Cysteamine: 5.97 Placebo: 5.2
Karrabi et al. [15].	2020	Iran	Randomized, double-blind, controlled	Cysteamine group (*n* = 25) MKF group (*n* = 25)	III–IV	Cysteamine: 35.0 MKF: 35.8	Cysteamine: 12.63 MKF: 12.3	2, 4	Cysteamine: 3.41 MKF: 3.31
Lima et al. [7].	2020	Brazil	Quasi-randomized, single-blind, controlled	Cysteamine group (*n* = 20) Hydroquinone group (*n* = 20)	II–V	Cysteamine: 42.0 Hydroquinone: 43.0	Cysteamine: 8.65 Hydroquinone: 5.43	2, 4	Cysteamine: 12 Hydroquinone: 14
Karrabi et al. [31].	2021	Iran	Randomized, single-blind, controlled	Cysteamine group (*n* = 27) Tranexamic acid group (*n* = 27)	III–IV	Cysteamine: 35.2 Tranexamic: 34.3	Cysteamine: 11.68 Tranexamic: 10.43	2, 4	Cysteamine: 3.61 Tranexamic: 3.54
Nguyen et al. [32].	2021	Australia	Randomized, double-blind, controlled	Cysteamine group (*n* = 10) Hydroquinone group (*n* = 10)	II–V	Cysteamine: 43.1 Hydroquinone: 44.2	Cysteamine: 7.1 Hydroquinone: 9.2	2, 4	Cysteamine: 5.5 † Hydroquinone: 7.0 †
Sepaskhah et al. [33].	2022	Iran	Randomized, single-blind, controlled	Cysteamine group (*n* = 39) hydroquinone/ascorbic acid combination group (*n* = 37)	NA	Cysteamine: 39.6 combination: 37.0	Cysteamine: 6.54 combination: 6.22	2, 4	Cysteamine: 6.94 combination: 6.08
Sachdev et al. [18].	2024	India	Randomized, double-blind, multi-arm-controlled	Cysteamine group (*n* = 30) Placebo group (*n* = 20) MKF group (*n* = 30)	III–V	Cysteamine: 42.6 Placebo: 42.0 MKF: 40.5	Cysteamine: 10.48 Placebo: 10.67 MKF: 10.51	1, 2, 4	NA

* Intention-to-treat population was recorded. Mansouri et al., Sepaskhah et al., and Sachdev et al. exclusively presented per-protocol data during the outcome assessment.; † median; SD, standard, deviation; MASI, Melasma Area Severity Index; MKF, modified Kligman’s formula; NA, not applicable.

**Table 2:** Regimens of included studies.

Study	Experimental Regimens	Controlled	Efficacy Assessment
Mansouri (2015) [8]	5% cysteamine, topical, at bedtime, daily *	Placebo	MASI, Mexameter^®^, IGA
Farshi (2018) [14]	5% cysteamine, topical, ar bedtime, daily *	Placebo	MASI, Mexameter^®^, Dermacatch^®^, IGA
Karrabi (2020) [15]	5% cysteamine, topical, in the evening, daily (15 min)	Modified Kligman’s formula	mMASI, IGA
Lima (2020) [7]	5% cysteamine, topical, at night, daily (up to 120 min)	4% hydroquinone	mMASI, Dif*L, GAIS
Karrabi (2021) [31]	5% cysteamine, topical, at bedtime, daily (30 min)	Tranexamic acid mesotherapy	mMASI, Dermacatch^®^
Nguyen (2021) [32]	5% cysteamine, topical, in the morning or evening, daily (15 min)	4% hydroquinone	mMASI
Sepaskhah (2022) [33]	5% cysteamine, topical, at night, daily (15 min)	Hydroquinone 4%/ascorbic acid 3% combination cream	mMASI, SkinColorCatch^®^ (Dermacatch^®^), IGA, PGA
Sachdev (2024) [18]	Cysteamine isobionic-amide, topical, in the evening, daily (15 min)	placebo, modified Kligman’s formula	mMASI, Dif*L, IGA

* No detailed cysteamine exposure time reported; MASI, Melasma Area Severity Index; mMASI, Modified Melasma Area Severity Index; IGA, Investigator’s Global Assessment; Dif*L, difference in colorimetric luminosity; GAIS, Global Aesthetic Improvement Scale; PGA, patient’s global assessment.

**Table 3:** Coefficients from meta-regression analyses.

Covariate	Coefficient	*p*-Value
Disease Duration	0.38	0.0001
Disease Duration *	0.23	0.32
Baseline MASI	−0.36	0.06
Age	0.21	0.16
Sample Size	−0.01	0.82

* Sensitivity analysis conducted by removing Lima et al.

## Data Availability

All data analyzed are cited and attached in this article. The protocol of the study was pre-registered on INPLASY (INPLASY202480016).
